# Editorial: The challenges of drug repurposing in diseases related to chronic inflammation

**DOI:** 10.3389/fphar.2023.1242880

**Published:** 2023-07-03

**Authors:** Debora Collotta, Laura Lucarini

**Affiliations:** ^1^ Department of Neurosciences “Rita Levi Montalcini”, University of Turin, Turin, Italy; ^2^ Department of Neurosciences, Psychology, Drug Research and Child Health (NEUROFARBA), University of Florence, Florence, Italy

**Keywords:** repurposing drugs, diet-related disease, metaflammation, cytokines, ages, metabolic disorders (MDs), inflammation, NLRP3 inflammasome

The process of inflammation is an intricate and essential response to biological, chemical, or physical stimuli. Ongoing research aims to unravel the cellular and molecular events that initiate and regulate the interactions among the various components involved in the inflammatory process. Numerous diseases, including arthritis, asthma, atherosclerosis, diabetes, cancer, and age-related conditions, have been associated with chronic inflammation.

To address the challenges of high attrition rates, significant costs, and slow pace of new drug discovery and development, it is crucial for current research to focus on repurposing existing drugs. Given the multiple pathways involved in the development of inflammation, drugs already available on the market for other therapeutic purposes that can intervene in resolving inflammation may hold potential for mitigating the impact and consequences of other disorders with similar underlying mechanisms. However, repurposing drugs presents challenges due to the need to identify suitable candidates that can effectively compete with the numerous drugs commonly used in clinical practice for specific diseases. Overcoming this hurdle requires extensive research.

The objective of this Research Topic was to gather research and review papers that demonstrate the significant interplay between inflammation and disease progression, as well as provide a deeper understanding of the molecular mechanisms involved in the assessment of repurposed drugs.

This Research Topic begins with an article by Xu et al., entitled “*Detecting potential mechanism of vitamin D in treating rheumatoid arthritis based on network pharmacology and molecular docking*”, delves into unraveling the potential mechanism by which vitamin D treats rheumatoid arthritis (RA). Vitamin D plays a pivotal role in RA; however, the mechanism of vitamin D in RA is still unclear. Therefore, this study employs network pharmacology and molecular docking strategies to investigate its mechanism. By examining 1,139 RA targets and 201 vitamin D targets, the researchers identify 76 overlapping genes. Enrichment analysis reveiled that cell proliferation, immune response, and apoptotic process are critical biological processes influenced by vitamin D in treating RA. The study also identifies antifolate resistance, osteoclast differentiation, and the nuclear factor-kappa B (NF-κB) signaling pathway as fundamental mechanisms involved in the treatment of RA with vitamin D. Further molecular docking analysis suggests that ALB, TNF, CASP3 and TP53 may serve as crucial checkpoints or diagnostic markers for future RA treatment.

Another noteworthy contribution to the Research Topic is a review article by Chaffey et al., titled “*Drug repurposing in cardiovascular inflammation: Successes, failures, and future opportunities*”. Drug repurposing is a pragmatic and appealing approach to drug discovery that has shown success in various medical fields over the years. Repurposing existing medications for new indications can significantly reduce development costs and timelines compared to *de novo* drug discovery process, making it a promising strategy in the field of cardiovascular disease, where new drug approvals lag behind other areas. Chronic inflammation has been established as a driving factor in cardiovascular disease pathology, leading to numerous attempts to therapeutically target cardiovascular inflammation. However, this approach has faced challenges, particularly off-target effects associated with broad-spectrum immunosuppression, which is especially problematic in long-term conditions like cardiovascular disease. Nevertheless, multiple anti-inflammatory drugs, repurposed from their original indications in autoimmune conditions such as rheumatoid arthritis, have been evaluated for efficacy in cardiovascular clinical trials. This review discusses the mixed outcomes of clinical trials investigating the use of anti-inflammatory drugs like anti-cytokine monoclonal antibodies, colchicine, and methotrexate in cardiovascular disease. Additionally, the authors highlight potential new directions for drug repurposing in cardiovascular inflammation, including the emerging concepts of drug re-engineering and chrono-pharmacology.

Song et al. published a review titled “*Natural drugs targeting inflammation pathways can be used to treat atherosclerosis*” focusing on the use of natural fdrugs for treating atherosclerosis (AS). AS involves the gradual degradation of arteries accompanied by inflammation While current research has primarily focused on the interactions among inflammatory cells, inflammatory mediators and immune mechanisms, some studies have reported the efficacy of natural drugs against AS. However, the utilization of natural drugs is often limited by factors such as poor penetration across biological barriers, low bioavailability, and unclear mechanisms.

In addition to the articles mentioned above, another valuable contribution to this Research Topic is the review article by Zhang et al., titled “*The emerging roles of TLR and cGAS signaling in tumorigenesis and progression of ovarian cancer*”. The authors highlight the significance of uncovering essential molecular biomarkers associated with the progression of ovarian cancer, considering its complex pathogenesis. Inflammation has been reported to play a crucial role in the initiation and progression of ovarian cancer, with both microenvironmental and tumor cell intrinsic inflammatory signals contributing to its malignancy. The review discusses the roles of receptors related to innate immune system, particularly Toll-like receptors (TLRs) and cyclic GMP-AMP synthase (cGAS), in the development and treatment of ovarian cancer. It sheds light on the intrinsic and exogenous inflammatory responses triggered by various stimuli and emphasizes the potential implications of these pathways in the management of ovarian cancer.

Finally, an important clinical trial article by El-Khateeb et al., entitled “*Evaluating the safety and efficacy of the leukotriene receptor antagonist montelukast as adjuvant therapy in obese patients with type 2 diabetes mellitus: A double-blind, randomized, placebo-controlled trial*” provides valuable insights into the treatment of obese patients with type 2 diabetes. The study aimed to evaluate the effectiveness, tolerability and safety of combining montelukast therapy with metformin in this patient population. The results demonstrated that the combination therapy of metformin and montelukast significantly improved diabetes control and induced weight loss. This improvement was attributed to the increased insulin sensitivity and anti-inflammatory properties of montelukast. The study concluded that the combination therapy was well-tolerated and safe throughout the duration of the trial. These findings suggest a potential role for montelukast as an adjuvant therapy in obese patients with type 2 diabetes.

In conclusion, the papers included in this Research Topic emphasize the strong relationship between inflammation and disease progression, providing a deeper understanding of the molecular mechanisms involved in the assessment of repurposed drugs. Additionally, they address concerns regarding potential adverse effects and pharmaceutical interactions associated with the use of repurposed drugs.

